# Aging of the hypothalamic-pituitary-adrenal axis in nonhuman primates with depression-like and aggressive behavior

**DOI:** 10.18632/aging.100227

**Published:** 2010-08-11

**Authors:** Nadezhda D. Goncharova, Victor Y. Marenin, Tamara E. Oganyan

**Affiliations:** Laboratory of Endocrinology, Research Institute of Medical Primatology of the Russian Academy of Medical Sciences, Sochi, Adler, Veseloye 1, 354376, Russian Federation

**Keywords:** stress, types of adaptive behavior, aging, Macaca mulatta

## Abstract

We have investigated aging of the hypothalamic-pituitary-adrenal (HPA) axis in female rhesus monkeys that differ in adaptive behavior. Plasma cortisol (F) and dehydroepiandrosterone sulfate (DHEA-S) concentrations under basal conditions and under acute psycho-emotional stress were evaluated in blood plasma of young (6-8 years) and old (20-27 years) female rhesus monkeys with various types of adaptive behavior (aggressive, depression-like, and average). We have found that the age-related changes in the HPA axis of monkeys with depression-like behavior were accompanied by the maximal absolute and relative hypercortisolemia under both basal conditions and stress. Moreover, young aggressive monkeys, in comparison with young monkeys of other behavior groups, demonstrated the highest plasma levels of DHEA-S and the lowest molar ratios between F and DHEA-S. Thus, age-related dysfunctions of the HPA axis are associated with adaptive behavior of animals.

## INTRODUCTION

The incidence of the stress-related diseases, such as depression, anxiety, insulin resistance, coronary heart disease, hypertension, cerebrovascular insufficiency, and some other diseases dramatically increases through ageing [for example, 1-6]. On the one hand, this may be conditioned by the increased stressfulness of this period of life due to the loss of physical health and social status, etc. However, it has been shown that intrinsic factors, contributing to decreased stress adaptability in old animals, namely disturbances of the hypothalamic-pituitary-adrenal (HPA) axis, are likely to be responsible, at least in part, for health deterioration in the aged [7-11]. Furthermore, there are individual peculiarities in vulnerability and resistance to stresses and stress-related pathologies among different persons due to the heterogeneity of the ageing process. Therefore, it is of great importance to elucidate the individual specificity of age-related changes of the HPA axis in the context of ageing of the organism as a whole and the stress-dependent age pathologies.

Studying the function of the HPA axis functioning under normal and stress conditions in individuals with various types of adaptive behavior is one approach to resolve this problem. Indeed there is evidence that links behavioral features of the individual with peculiarities in their HPA axis function [9;12-15]. Hyperactivation of the HPA axis occurs in persons who exhibit increased anxiety, in those with some forms of depression [14-17] as well as in animals with depression-like behavior [12,18,19]. Moreover, there are data showing a decline in dehydro-epiandrosterone (DHEA) and dehydroepiandrosterone sulfate (DHEA-S) secretion in depressed persons [20, 21]. On the contrary, DHEA-S is elevated in individuals exhibiting aggressive behavior [22-24].

However, there are no data on the individual specificity of the age-related changes within the HPA axis, namely data on how the functioning of the HPA axis varies through ageing in individuals with different types of adaptive behavior. Nonhuman (laboratory) primates are the preferable model for this type of research. Indeed, nonhuman primates and humans are similar in their diurnal pattern of the HPA axis activity, i.e. high activity during daylight hours and low activity during the night. Furthermore, cortisol (F) is the main glucocorticoid hormone both for non-human primates and humans. Moreover, primates are unique animal models in which adrenal DHEA-S secretion undergoes a decline with ageing [25-32].

Because DHEA-S may exhibit antiglucocorticoid properties [33-35] some argue that, in primates, the molar ratio of F to DHEA-S (F/DHEA-S coefficient) is a better measure of hypercortisolemia than F alone [36-38]. Furthermore, unlike rodents, nonhuman primates feature the psycho-emotional reactions and adaptive behavior more similar to those in humans [39-41].

The purpose of this work was to investigate whether there are differences in the HPA axis function in young and old female rhesus monkeys of various behavioral types, under basal conditions, as well as under conditions of acute psycho-emotional stress.

## RESULTS

### Activity of the HPA axis in young and old female rhesus monkeys with different types of behavior under basal conditions

The data presented in Table 1 show the basal levels of F in blood plasma of young and old female rhesus monkeys with different types of behavior. Neither inter-group, nor age-related differences proved to be statistically significant. The absence of age-related differences in F levels at 0900 hours was confirmed by correlation analysis. The correlation coefficient (r) was -0.14 for animals with aggressive behavior, -0.20 for animals with depression-like behavior, and -0.25 for animals with average behavior.

**Table 1.** Plasma cortisol levels in young and old female rhesus monkeys with different types of behavior under basal conditions (mean ± S.E.M.)

Age, years	F, nmol/l		
	Types of behavior (groups of animals)		
	Aggressive, n = 8 (group 1) n = 8	Depression-like, n = 13 (group 2)	Average, n = 13 (group 3)
6-8	879 ± 70	984 ± 50	935 ± 40
20-27	825 ± 60	916 ± 50	850 ± 40

Table 2 shows the plasma levels of DHEA-S for young and old female rhesus monkeys of different behavioral types. For young animals, one can see significantly higher levels of DHEAS in aggressive animals in comparison with animals with an average type of behavior or depression-like behavior. For old animals, however, no statistically significant inter-group differences in DHEA-S concentrations were observed. Thus DHEA-S concentrations in old animals of all behavioral groups, including animals with aggressive behavior, were considerably lower in comparison with those in young animals of respective groups. It is also worthy of note that DHEA-S concentrations underwent maximum age-related changes in the first group of animals (5.07-fold), and minimal age-related changes in the third group (1.95-fold). In old animals with depression-like behavior, concentrations of DHEA-S decreased, on average, 2.56-fold. Correlation analysis revealed a negative correlation between DHEA-S levels and age in all behavioral groups. The correlation coefficient was -0.58 for animals with aggressive and depression-like behavior and -0.59 for animals with average behavior.

**Table 2.** Plasma DHEA-S levels in young and old female rhesus monkeys with different types of behavior under basal conditions (mean ± S.E.M.)

Age, years	DHEA-S, nmol/l		
	Types of behavior (groups of animals)		
	Aggressive, n = 8 (group 1)	Depression-like, n = 13 (group 2) (группа 2)	Average, n = 13 (group 3)
6-8	1370 ± 260	630 ± 80 ●	640 ± 70 ●●
20-27	270 ± 40***	246 ± 50***	328 ± 40***

*** P < 0.001 - age-related differences;

●● P < 0.05; ●● P < 0.01 - vs. young aggressive animals.

**Figure 1.**
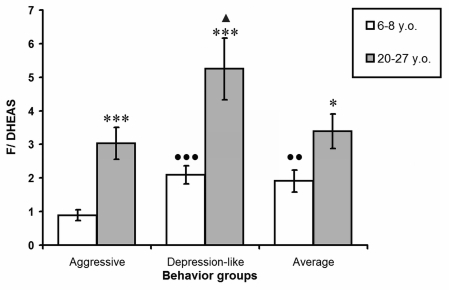
The molar ratio between F and DHEA-S plasma levels in young and old female rhesus monkeys with different types of behavior (mean ± S.E.M.) * P < 0.05; *** P < 0.001 - age-related changes; ●● P < 0.01; ●● P < 0.001 - vs. young aggressive animals; ▲ P < 0.05 - vs. old aggressive animals.

Figure 1 represents the molar ratios between F and DHEA-S levels (F/DHEA-S coefficient) in young and old female rhesus monkeys with different behavioral types. The lowest values for this coefficient in young animals with aggressive behavior significantly differed from those in animals of other behavioral groups. With ageing, the F/DHEA-S coefficient increased significantly in animals of all investigated groups. However, the highest values for this ratio were marked in old monkeys with depression-like behavior, and were significantly higher in comparison with those in animals with aggressive behavior. Furthermore in old aggressive animals the values of the molar ratios between F and DHEA-S levels equalized with those seen in old animals with average behavior, despite the minimal values for this coefficient in young aggressive animals.

The obligate increase in F/DHEA-S coefficient values during ageing was confirmed to strongly correlate with the age of animals in all behavioural groups. The correlation coefficient was 0.75 for animals with aggressive behavior, 0.68 for animals with depression-like behavior, and 0.65 for animals with average behavior.

The data presented in Table 3 show diurnal changes in plasma F levels for young and old animals with different behavioral types. For young animals, one can see similar circadian rhythms of F in all behavior groups. During ageing the amplitude of circadian rhythm of F significantly decreased in monkeys with depression-like behavior, mainly due to an increase in F concentrations at 2100 hours. F levels in old animals in the second group at 2100 hours were significantly higher in comparison with those in young monkeys in the same group (747 ± 50 and 567 ± 50 nmol/l, respectively, p < 0.05). However F levels in this group did not significantly differ between old and young animals in the morning (0900 hours) (911 ± 70 and 1011 ± 30 nmol/l, respectively, p > 0.05).

**Table 3.** Plasma cortisol levels at different time of day and the amplitude of circadian rhythm of cortisol levels in young and old female rhesus monkeys with different types of behavior (mean ± S.E.M.)

Age, years	Time of day, hours	Types of behavior (groups of animals)		
		Aggressive, n = 6 (group 1)	Depression-like, n = 11 (group 2)	Average, n = 11 (group 3)
		F, nmol/l		
6-8	0900h	955±120	1011±30	911±40
	2100h	547±100 ♦	567±50 ♦♦	475±70 ♦♦
	Amplitude	408±120	444±80	436±50
20-27	0900h	714±100	911±70	825±90
	2100h	220±76 * ♦ ♥♥	747±50 *	408±70 ♦♦ ♥
	Amplitude	494±70 ♥	164±80 *	417±50 ♥

* P < 0.05 - age-related differences;

▲ P < 0.05; ♦♦ P < 0.001 - vs. the relative values at 0900h;

♥ P < 0.05; ♥♥ P < 0.01 - vs. the relative values for group 2.

A correlation analysis indicated that the age-related changes in the amplitude of the F circadian rhythm were mainly due to the age-related changes in F levels in the evening (2100 hours). The correlation coefficient for F levels at 2100 hours and the amplitude of the F circadian rhythm in monkeys with depression-like behavior, irrespective of age was -0.70, while that for F levels at 0900 hours and the amplitude of the F circadian rhythm was -0.40.

The amplitude of the F circadian rhythm did not change with age in monkeys with aggressive and average behavior. Age-related changes in the amplitude of the circadian F rhythm in animals of the second group gave rise to significant inter-group distinctions in the amplitude of F in old animals, with minimal values in animals with depression-like behavior.

### Activity of the HPA axis in young and old female rhesus monkeys with different types of behavior under conditions of acute moderate psycho-emotional stress

**Figure 2.**
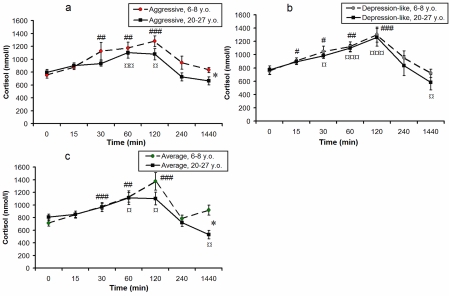
Dynamics of plasma F concentrations in response to acute psycho-emotional stress for young and old female rhesus monkeys with different types of behavior (mean ± S.E.M., nmol/l). * P < 0.05 - age-related differences; # P < 0.05; ## P < 0.01; ### P < 0.001 - vs. the relative values before stress (0 min) for young animals; ¤ P < 0.05; ¤¤ P < 0.01; ¤¤¤ P < 0.001 - vs. the relative values before stress (0 min) for old animals.

Figure 2 shows the dynamics of F levels in response to acute psycho-emotional stress for young and old monkeys with different types of behavior. In young monkeys the minimal significant increase of plasma F levels was observed 30 minutes after the initiation of immobilization, and the maximum - 120 minutes after the initiation of stress. Plasma F levels decreased in all groups through the 4-hour period after stress initiation These returned to initial baseline levels only 24 hours after the stress.

In old monkeys the dynamics of F levels in response to acute psycho-emotional stress had, on the whole, a similar character to the relative changes in young animals of respective behavioral groups. However the maximum concentrations of F in old animals with aggressive and average types of behavior (120 minutes after initiation of immobilization) showed the tendency to decrease in comparison with young animals, and returned to initial values 4 hours after the onset of restraint. Twenty-four hours after stress, F levels were lower than basal values in old animals of all groups. Twenty-four hours after stress, F levels in old animals with aggressive and average types of behavior were significantly lower in comparison with young animals of respective behavioral groups (p < 0.05).

**Figure 3.**
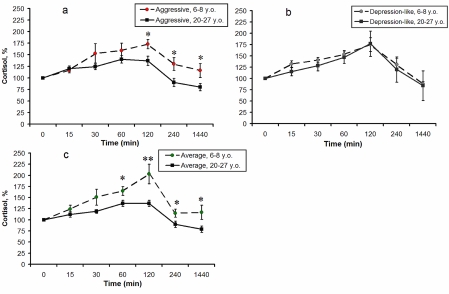
Dynamics of plasma F concentrations in response to acute psycho-emotional stress for young and old female rhesus monkeys with different types of behavior (mean ± S.E.M., % of basal level). * P < 0.05; ** P < 0.01 - age-related differences.

Additionally, in comparison with young monkeys, old animals of the first and third groups demonstrated a significantly lower increase (expressed as percentage increase) in F levels in response to immobilization, 120 minutes after stress initiation (p < 0.05 for aggressive animals; p < 0.01 for animals with average behavior, see Figure 3a, 3c). Besides, old animals with average behavior demonstrated a significantly lower increase in F concentrations in response to stress 60, 240 minutes and 24 hours after the onset of stress (Figure 3c), and old animals with aggressive behavior demonstrated a significantly lower increase of F 4 and 24 hours after onset of immobilization (Figure 3a). These data suggest a decrease in stress responsiveness of the HPA axis with ageing in animals with aggressive and average types of adaptive behavior. However, no significant age-related changes in plasma F were revealed for animals with depression-like behavior (Figure 3b).

## DISCUSSION

We evaluated the behavior of monkeys housed individually, in metabolic cages. During the 2-week period immediately following the transfer of monkeys from their usual residence, in groups, into the individual metabolic cages, they exhibited considerable orientation and aggressive-defensive unconditioned reflexes in response to new living conditions, as well as to the procedure of bleeding. A 4-week period was sufficient for the elimination of these reflexes in animals with average (standard) behavior, but not in animals with aggressive and depression-like behavior. Behavior following the adaptation period, i.e. during experimentation was distinctly different between groups, and we considered this to be each animals' individual response to mild stress induced by the procedure of bleeding (procedural stress), but not due to housing in metabolic cages, since we considered the 4-week adaptation period spent in metabolic cages to be a training procedure. Our data on the polymorphism of the behavioral response of laboratory primates to mild stress are confirmed by data from other authors [44,45].

We have shown that under basal conditions, at 0900 hours, F concentrations did not undergo considerable inter-group changes, neither in young, nor in old animals (Table 1). But basal F concentrations at 2100 hours in old animals with depression-like behavior was significantly higher in comparison with those in young animals of the same behavioral group, and are also much higher than those in old animals of other behavioral groups (Table 3). An increase in F levels at 2100 hours signifies the development of hypercortisolemia in old monkeys with depression-like behavior, since F levels are much higher in these animals compared with those in old animals with aggressive and average behavior.

At the same time, concentrations of DHEA-S, another important corticosteroid and regulator of F activity, underwent considerable inter-group differences in young and old monkeys under basal conditions. Concentrations of DHEA-S in aggressive young animals was significantly higher in comparison with those in young animals of other behavioral groups, while DHEA-S concentrations in old animals with depression-like behavior tended to be lower than those in old animals with average behavior (Table 2).

Our data on higher basal concentrations of DHEA-S in young animals with aggressive behavior (Table 2) are consistent with results from clinical research, which showed an increase in DHEA-S plasma levels in persons with increased aggression [22-24]. Augmented levels of circulating DHEA-S in primates can be metabolized into active sex steroids (e.g. testosterone) within various peripheral tissues, including the brain [46,47], and/or can inhibit GABA_A_ receptors [19,48], that, in any case, can be the reason of aggressive behavior. So, it is well-known that testosterone mediates aggression in a wide range of species, including humans [47,49,50]. On the other hand, inhibition of GABA_A_ receptors enhances aggressive behavior [51,52].

With ageing, a drastic decrease in the levels of DHEA-S in peripheral blood of monkeys of all behavioral groups took place. It is interesting that DHEA-S concentrations did not significantly differ with behavioral type in old animals despite significant behavior-related differences in concentrations of this corticosteroid in young animals (Table 2). Similar changes, arising from the age-related attenuation in DHEA and DHEA-S secretion by the adrenal cortex, have already been observed through experiments with monkeys of various species [26,31,53-58] and humans [26,37,59-61].

Basal DHEA-S but not F concentrations at 0900 hours showed marked inter-group and age-related differences. Therefore we considered it important to calculate the F/DHEA-S coefficient for animals of all behavioral groups, both young and old, under basal conditions at 0900 hours. A growing body of research has provided evidencethat DHEA-S exhibits antiglucocorticoid properties [33-35]: it is possible that the F/DHEA-S molar ratio mayindex the degree to which an individual is buffered againstthe negative effects of stress [38,62,63], and may therefore be considered as one of the biomarkers of ageing [32,36,55,64].

The F/DHEA-S ratio in young animals was at its lowest in the aggressive group owing to the highest levels of DHEA-S in this group (Figure 1). The F/DHEA-S coefficient values were markedly greater in old animals than in young ones, irrespective of behavior type. Furthermore, while F/DHEA-S coefficient values did not significantly differ between old aggressive animals and those with average behavior, values for this ratio in old animals with depression-like behavior were significantly higher in comparison with old animals with aggressive and average behavior. The high F/DHEA-S ratio values in old monkeys serve as evidence for the development of relative hypercortisolemia at 0900 hours under basal conditions.

The increase in F/DHEA-S ratio values with ageing has been reported in several nonhuman primate species, including Papio hamadryas [53-55], Macaca mulatta [54-57]; Macaca nemestrina [31], as well as in the human, during physiological ageing, and to a greater extent - in dementia [28,32,36,37,64-66].

It is important to note that many authors have shown a positive correlation between F levels or F/DHEA-S ratio values and the incidence of morphometry disorders of brain structures under corticosteroid regulation (e.g. hippocampus) in some physiological and pathological conditions, such as ageing, neurodegenerative diseases, posttraumatic stress disorder and depression [9,36,64,67,68]. Of particular interest is the observation that a reduction in the volume of the hippocampus in old persons (as visualized using magnetic resonance imaging) is accompanied by increase in F and subsequently in F/DHEA-S ratio values [9,36,64,69]. The hippocampus is a critical area for learning and memory, as well as for the regulation of the HPA axis, with high levels of glucocorticoid receptor expression [9,70-72]. It is widely accepted that glucocorticoid hormones and adrenal androgens have effects on the hippocampus [11,33,34]. The effects of the two corticosteroids on the hippocampus are antagonistic: excess F promotes neuronal degeneration and death, whereas DHEA-S plays a neuroprotective role with regards to prevention of structural damage and functional impairment, owing to physiological ageing or pathological condition, and stimulates neuronal survival [9,11,33,34,69]. Thus, the age-related increase in F/DHEA-S molar ratio values is invariably associated with impairment in DHEA-S-mediated antiglucocorticoid activity, and consequently in enhanc-ed glucocorticoid neurotoxicity.

The highest F/DHEA-S ratio in circulation was in old monkeys with depression-like behavior (Figure 1), which is in agreement with clinical data from patients with some forms of depression [13,14,20,21]. This suggests that an imbalance in the ratio of F to DHEA-S may be causative of the depression-like condition in monkeys with depression-like behavior. This is consistent with data from Goodyer et al. [38], who had shown that circulatory F/DHEA-S molar ratio values, but not F levels alone, are an effective correlate of hypercortisolemia in patients with depression. Furthermore, it has been revealed that enhanced blink reflex expression, in response to a common stimulus, is characteristic in persons with increased anxiety, and the severity of their anxiety positively correlates with F/DHEA-S molar ratio values [73]. It is hardly surprising, therefore, that administration of DHEA reduces depressive symptoms [34,74].

The decrease in the amplitude of the F circadian rhythm has been consistently reported in studies of human and laboratory primate ageing, and this evidence has allowed many researchers to consider this parameter as one of the biomarkers of ageing [32,36,75,76]. However, our study of age-related changes in the amplitude of the F circadian rhythm in female rhesus monkeys, in relation to features of adaptive behavior, has allowed us to reveal a statistically significant decrease in this correlate (due to an increase in F concentrations at 2100 hours) only in monkeys with depression-like behavior (Table 3). It is of note that high evening F levels were recorded in adolescents with depressive symptoms [77,78] and in adult patients with major depressive disorder [15]. Perhaps ageing in healthy individuals with depression-like behavior is accompanied by more expressed disturbances in the diurnal fluctuations within the HPA axis.

Age-related differences in the stress responsiveness of the HPA axis in monkeys with aggressive and average types of behavior (Figure 2a, 2c; Figure 3a, 3c) are consistent with results from our previous studies, in which specific features of adaptive behavior of animals were not considered, but have shown lower stress responsiveness of the HPA axis in old female rhesus monkeys, in comparison with young animals, in the afternoon [8,79]. Meanwhile there was a weaker tendency for HPA axis stress responsiveness to decrease in old animals with depression-like behavior, in comparison with young animals of the same behavioral type (Figure 2b, 3b).

The fact that there were no significant age-related changes in the stress responsiveness of the HPA-axis in monkeys with depression-like behavior is unlikely to be indicative of an enhanced resistance to the effects of stress and ageing in these animals. On the contrary, to some extent the values of the biomarkers of aging (i.e. plasma DHEA-S levels, the molar ratio of F to DHEA-S, and the amplitude of the F circadian rhythm) indicate more pronounced age-related changes in the function of the HPA axis in monkeys with depression-like behavior. Most likely, the absence of age-related changes in the extent to which F levels increase in response to the acute stress, in animals with depression-like behavior, indicates a more conspicuous stress-induced hypercortisolemia in these animals, as compared with animals of other behavioral types.

In conclusion, we have found that female rhesus monkeys with different patterns of adaptive behavior demonstrate similar age-related changes in the activity of the HPA axis accompanied by absolute and/or relative hypercortisolemia. Furthermore, we have shown that monkeys of various behavioral types are subjected to various severities of age-related disturbance in HPA axis activity.

We have found, for the first time, that monkeys with depression-like behavior demonstrate age-related changes in HPA axis function that are accompanied by maximal absolute and relative hypercortisolemia under basal conditions, as well as by a significantly greater increase in plasma F levels under acute stress. We have also found that young monkeys with aggressive behavior have higher levels of DHEA-S and lower molar ratios between plasma levels of F and DHEA-S as compared with young monkeys of other behavior groups. Such inter-group differences were not exhibited by old animals with aggressive behavior. Furthermore, minimal age-related changes in the HPA axis have been observed in monkeys with average behavior.

## METHODS

### Animals

Thirty-four young adult (6-8 years) and thirty-four old (20-27 years) healthy female rhesus monkeys (*Macaca mulatta*) were used in the experiments. The monkeys originated from the Research Institute of Medical Primatology, Sochi, Russia. The animals were housed in open enclosures or cages designed for group housing. During the observation period the animals were transferred into individual metabolic cages in a separate room with natural illumination (approximately from 06.00 to 18.00) and ambient surrounding temperature (from 20^°^C to 28^°^C). All experiments were carried out in the period of June-August when ovarian cycles are not typical for female rhesus monkeys. The animals were fed pellets prepared in the Institute according to the technique of Altromin Spezialfutter GmbH & Co. KG (Lage, Germany). The pellet diet was complemented with bread, boiled eggs, and fresh vegetables and fruit. Water was available ad libitum. The animals were adapted to living in metabolic cages and to the procedure of bleeding for 4 weeks before the experiments.

The animals' behavior was categorized while they were housed in metabolic cages, both during the period of adaptation, and throughout experimentation. Classification of behavior was done according to recommendations for laboratory primates [41,42]. Depending on behavioral features, both young and old animals were divided into three groups: the first groups comprised animals with active, aggressive type of behavior (8 young adult, 6.5 ± 0.3 years, 5.7 ± 0.6 kg; 8 old, 22.0 ± 0.4 years, 5.7 ± 0.3 kg); the second groups consisted of the animals mainly with passive, depression-like behavior (13 young adult, 6.9 ± 0.2 years, 5.0 ± 0.2 kg; 13 old, 22.2 ± 0.4 years, 5.1 ± 0.2 kg); the third groups included animals with active behavior, lacking strong features of aggression or depression-like behaviors (13 young adult, 6.9 ± 0.2 years, 5.3 ± 0.2 kg; 13 old, 22.5 ± 0.4 years, 5.7 ± 0.2 kg), and was thus referred to as the ‘average’ behavior group.

Animals fitting the first groups were identified by excessive aggressive-defensive reactions directed at researchers and the procedure of bleeding (e.g., demonstration of threat by means of glaring, yawning, barking, various poses of readiness to jump toward the experimenter, or to attack the metabolic cage construction, etc.), which persisted well beyond the four-week adaptation period, right through to the end of the experiment. Animals of this group always tried to compromise the procedure of bleeding, and snatched food reinforcements (fruit, cookies, sweets), used for attenuation of the defensive reflex following the blood sampling procedure.

Animals of the second groups were characterized by a typical exaggerated avoidance of the experimenters, which persisted throughout the adaptation period, as well as through the rest of the experiment. These monkeys were identified by prolonged periods of inactivity, hunched poses, attempts to hide in corners, and apathy towards the experimenter. These monkeys always tried to interfere with blood sampling by positioning themselves in a way that allows them minimally contact with the experimenter (head downwards, back to the experimenter) and did not react to food reinforcement in presence of experimenters.

The initial reactions of the animals in the third groups to experimenter (movements of the head and eyes in the direction of the stimulus, lying without movement or moving toward researcher) disappeared within the 2-4 weeks of the adaptation period. Following this period, these animals willingly came into a contact with researchers, were friendly, sat mainly towards the front of the cage, cooperated during blood sampling, and calmly accepted food reinforcements.

### Methods

For investigation of basal activity of the HPA axis all young [34] and old [34] animals, irrespective of behavioral category, underwent bleeding at 0900 hours. Levels of F and DHEA-S were measured in blood plasma and the molar ratio between F and DHEA-S levels (coefficient F/DHEA-S) was calculated. Additionally, in 28 animals from each age group with different behavioral type (6 animals for each age group with aggressive behavior, 11 - for each age group with depressive-like behavior and 11 - for each age group with average behavior) but on the other day blood samples were taken at 0900 and 2100 hours. The concentrations of F were measured in the plasma fraction of these blood samples and the amplitude of circadian rhythm of F was calculated.

Animals of all age and behavioral groups underwent an acute stress procedure: moderate restraint in a metabolic cage for two hours. Restraint was achieved by using a conventional squeeze board to press the animal to the front wall of the metabolic cage. The body and extremities of the animal were not tightly immobilized. Animals were subjected to the stressor at 1500 hours. Blood samples were taken before immobilization (0), at 30, 60, and 120 minutes during application of the stressor, and at 240 minutes, i.e., 2 hours after termination of the stressor. Further blood samples were taken at 24 hours after onset of immobilization. At each time point, 2.5-3.0 ml of blood was taken. The plasma fractions of the blood samples were used for measurement of F.

All blood samples were taken from the cubital or femoral vein of the animals, which were fasted overnight, with heparin ("Sintez", Kurgan, Russia, 10 μl per 3 ml of blood) as the anticoagulant. Blood samples were immediately centrifuged at 2000 x *g* at +4^o^C. The plasma was separated and stored at - 70^o^C. F and DHEA-S in the plasma were determined by ELIZA using standard commercial kits (AlkorBio Ltd., St. Petersburg, Russia for F; DSL, Webster, TX, USA for DHEA-S). The intra-assay variation coefficients for F and DHEA-S (C.V.) did not exceed 10% (mean C.V. for F = 4.8; for DHEA-S = 6.3) and inter-assay variation coefficients did not exceed 15% (mean C.V. for F = 10.8; for DHEA-S = 8.8). To evaluate the circadian rhythm of plasma F concentration, the amplitude of circadian rhythm of F was calculated as the difference between the F concentrations at 0900 and at 2100 hours.

The experimental values are presented in tables and figures as means ± S.E.M. The statistical comparisons of the age, behavioral group, circadian differences in basal conditions and under restraint stress were performed using one- and two-way analysis of variances (ANOVA) including post hoc Dunnett's honest significant difference test for paired comparisons and correlation analysis where it is necessary [43].
